# Inferior Vena Cava and Renal Vein Thrombosis Associated with Thymic Carcinoma

**DOI:** 10.1155/2017/1793952

**Published:** 2017-01-10

**Authors:** Vlad Teodor Berbecar, Roxana Jurubita, Marina Paraschiv, Bogdan Obrisca, Bogdan Sorohan, Gener Ismail

**Affiliations:** ^1^University of Medicine and Pharmacy “Carol Davila”, Bucharest, Romania; ^2^Fundeni Clinical Institute, Bucharest, Romania

## Abstract

Thymic tumors are rare mediastinal tumors that can present with a wide variety of symptoms. They can cause distant manifestations and are frequently associated with paraneoplastic syndromes. In our case, we describe the evolution of a 68-year-old male whose first manifestation was thrombosis of the inferior vena cava and renal veins. Thrombosis of large abdominal veins is rare, especially without being associated with any other comorbidity or risk factors.

## 1. Introduction

Thymic tumors are rare mediastinal tumors that are considered orphan diseases due to their low prevalence. In the United States, they have an overall incidence of 0.13 cases per 100,000 person-year [1]. The most recent histologic classification divides thymic tumors into thymomas, thymic carcinomas (TC), and neuroendocrine thymic tumors (NETT) [2]. Thymic tumors show a variable and unpredictable evolution, ranging from a benign form that can slowly grow for years before being discovered to a rapidly evolving metastatic tumor. They can often be asymptomatic, or the patient can present with local symptoms: chest pain, cough, and shortness of breath. When the tumor extends beyond its capsule and invades the surrounding tissue, then more severe symptoms can occur, such as superior vena cava (SVC) syndrome, hemidiaphragm paralysis (phrenic nerve involvement), and hoarseness (recurrent laryngeal nerve infiltration).

Thymic tumors are often associated with paraneoplastic syndromes, with myasthenia gravis being the most frequent [3, 4]. The list is long; however, one of the rarer manifestations can include a hypercoagulability state that can lead to thrombosis of the adjacent veins or even distal veins [5, 6]. Abdominal vein thrombosis is a rare but potentially life-threatening form of venous thrombosis. Both inherited and acquired thrombophilia factors are frequently identified in cases of large vein thrombosis [7]. Advancements in molecular and genetic mechanisms have increased the spectrum of markers associated with a high risk of thrombophilia: mutation of FV Leiden 506R/Q, mutation of prothrombin (FII) 20210G/A, mutation of plasminogen activator inhibitor (PAI-1) 4G/5G, and others [8].

When a patient is diagnosed with a mediastinal mass, the differential diagnosis must include thymoma, lymphoma, thymic carcinoma, thymolipoma, germ cell tumors, and lung metastases but also thymic cysts, lymphangiomas, and aortic aneurysms [9, 10].

## 2. Case Presentation

We present the case of a 68-year-old male who is admitted to the regional hospital reporting pain in the upper abdomen.

The patient had no known history of any illness or any surgical intervention and did not take any medication at home. He was an ex-smoker (20 packs/year) but had quit smoking almost 25 years ago. His complaints started approximately a week before and progressively worsened until he decided to go to the hospital.

The initial bloodwork showed slightly enhanced amylase = 174 U/L (*N* = 25–100 U/L) and lipase = 97 U/L (*N* = 0–60 U/L). EKG and chest radiography showed no modifications; however, the abdominal ultrasound revealed a right renal mass with modifications of the renal parenchyma and also modification of the pancreas ecostructure. The patient is transferred to our clinic with suspicion of right renal mass and pancreatitis.

The* physical examination* did not reveal much. The patient was in a relatively good state, with his only complaints being epigastric pain. His skin and mucosae were relatively pale, and he had telangiectasias on his face and chest. His respiration was normal, with 18 resp/min, and there were no audible rales. His blood pressure was 150/80 mmHg with 84 beats/min. His abdomen was slender and he reported slight pain on examination of the epigastrium.

The patient's* bloodwork* showed signs of anemia and thrombopenia which, in the 4th day after admission, reached the lowest level: 82.000/ul thrombocytes (*N* = 150–400.000/ul) and 11.1 g/dl of hemoglobin (*N* = 12–16 g/dl). He also had hypoalbuminemia, 3.1 g/dl (*N* = 3.5–5.1 g/dl), and proteinuria of 2.28 g/24 h.

**Figure 1:**
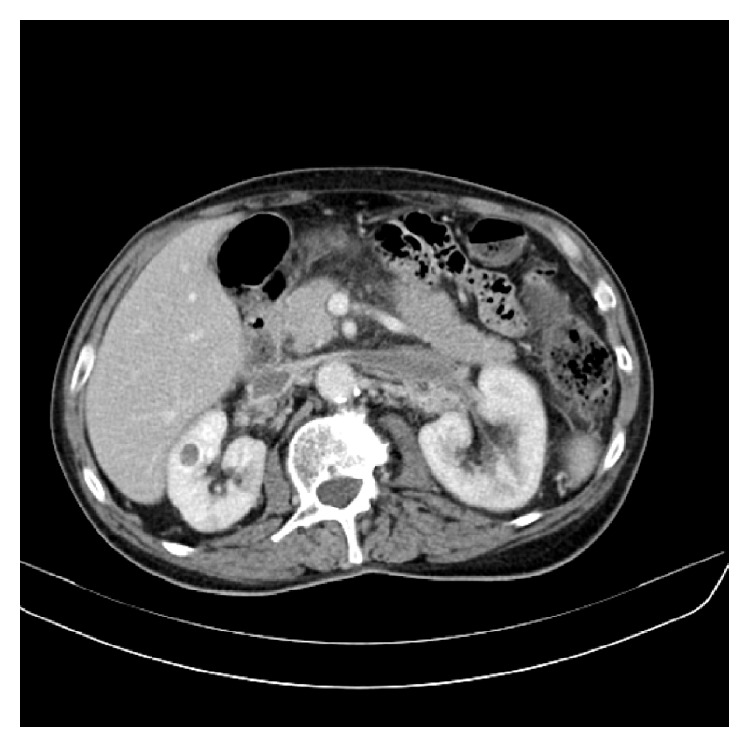
CT scan cross section: thrombosis of the left and right renal veins and of the portal vein.

The CT scan of the abdomen and pelvis proved to be an indispensable tool for the diagnosis. It showed thrombosis of the left and right renal veins and of the portal vein (Figure 1) with enlarged kidneys. Also multiple perigastric, celiomesenteric, and hepatic adenopathies were described. He was immediately started on i.v. heparin which afterwards was replaced with oral anticoagulants.

The abdominal CT excluded the suspicion of a renal tumor. Having ruled out the most likely suspect that can cause renal vein thrombosis, we started to look for more unusual causes. All the tumor markers came back negative. But the extended coagulation testes revealed elevated D-dimers (980 *µ*g), present PDF, and a* protein S deficiency* (45%). However, the protein S deficiency alone is not enough to cause the massive vein thrombosis. We also tested for a genetic hypercoagulability state, which included tests for factors II, V, MTHFR, and PAI genes; all tests came back negative.

**Figure 2:**
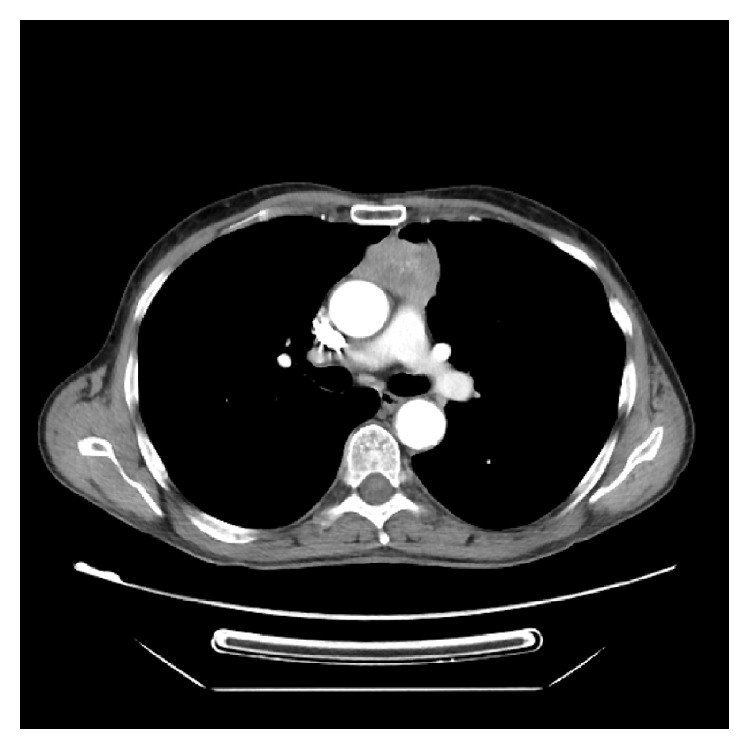
CT scan cross section: anterior mediastinal mass, upper quadrant, with irregular shape, heterogenous iodophilia; it has small areas of necrosis; axial dimension approx 49/29 mm and 50 mm craniocaudal; it is situated between the ascendant aorta and the trunk of the pulmonary artery and has lateral relation with the pulmonary parenchyma.

A thoracic CT scan was performed which revealed an* anterior mediastinal mass* in the upper quadrant, of approx 49/29 mm dimensions, with irregular shape and heterogenous iodophilia; it had small areas of necrosis; it was situated between the ascendant aorta and the trunk of the pulmonary artery and had lateral relation with the pulmonary parenchyma. Multiple mediastinal adenopathies could be seen (Figure 2).

The patient was scheduled for surgical intervention. Sternotomy was performed followed by tumorectomy of the anterior mediastinal mass and thymomectomy of the remaining thymus tissue. The thymic tumor mass was 7/5/3 cm in size and was encapsulated being well differentiated from the surrounding tissue. Histopathology showed an* undifferentiated carcinoma probably of thymic origin* (optical microscopy showed poorly differentiated carcinoma cells with frequent atypical mitosis in the thymic mass which was covered by a hyaline capsule; the immunohistochemistry showed a CD5 positivity along with negative TTF1, negative PSA, negative Ck7, negative CROMO, negative SYNAPTO, and positive CK34BetaE12, P63, and PSMA). The operation was uneventful and the patient remained under observation for almost two weeks.

**Figure 3:**
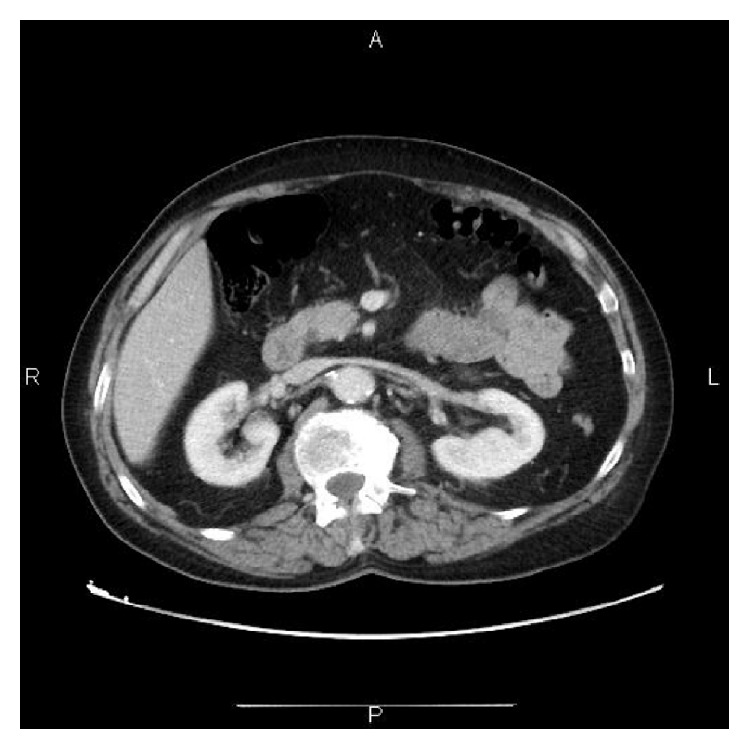
CT scan cross section: complete repermeabilisation of the inferior vena cava and of the renal veins.

When he returned a month later for a follow-up, he was symptom-free and was feeling like a new man. The dyspnea and abdominal pain were gone and he was no longer feeling fatigued. An abdominal ultrasound revealed partial regression of the inferior vena cava thrombosis. The patient remained on oral anticoagulants and was followed up every 3 months. After one year, a control CT scan showed complete regression of the inferior vena cava and renal vein thrombosis (Figure 3). He was symptom-free and there was no need to continue taking acenocumarol.

## 3. Discussion

Thymic tumors are rare mediastinal tumors and, most often, they are discovered by using imaging techniques (CT) because of nonspecific symptoms. However, in our case, thrombosis of the inferior vena cava and of both renal veins as the first manifestation of such tumors has never been described in the literature. The nonspecific symptoms of the patient (asthenia, dry cough, and pain in the upper abdomen) were what determined us to closely investigate the patient and perform an echography and, afterwards, a CT scan. But to our surprise, the cause of his symptomatology was neither abdominal nor pulmonary but mediastinal. The thymic carcinoma manifested itself as a paraneoplastic syndrome which induced a hypercoagulability state that led to the thrombosis of the inferior vena cava and renal veins. The modifications in the patient's blood can be attributed to the thrombosis which affected the connected organs (causing proteinuria, increased amylase), except for the anemia and the thrombopenia which might have been another paraneoplastic manifestation. The patient's hereditary thrombophilia (the protein S deficiency) also played an important part and was probably the reason why the first paraneoplastic manifestation presented as a thrombosis of major veins.

The initial therapeutic attitude after the diagnosis of inferior vena cava and renal vein thrombosis was the correct one. Firstly, heparin anticoagulation was initiated and was maintained until oral anticoagulants could be administered and had taken effect. This prevented the thrombosis from advancing and even managed to slightly regress in its spread. The thoracic surgeon performed ideal tumorectomy and according to the Masaoka-Koga staging system (1994), the thymic tumor was of stage I (grossly and microscopically completely encapsulated tumor) [11]. For this reason, no adjuvant chemotherapy or radiotherapy was necessary [12–14].

One month after the excision of the tumor, the thrombosis of the inferior vena cava and of the renal veins had decreased (on Doppler echography). The symptomatology had completely disappeared and the patient remained on oral anticoagulants. He was followed up every 3 months and was scheduled for a CT scan after one year. When the follow-up CT scan was performed it showed complete regression of the inferior vena cava and renal vein thrombosis. Being symptom-free and with no sign of thrombosis, the patient was taken of oral anticoagulants.

## 4. Conclusion

Thrombosis of the inferior vena cava and renal veins is a rare manifestation that can be caused by a handful of diseases. The list is short, with the most frequent being renal cancer. In our case, it was a manifestation of a paraneoplastic syndrome caused by thymic carcinoma. Although rare, thymic carcinomas can cause distant manifestations and are discovered when nonspecific symptoms start to emerge. In our case, the inferior vena cava and renal vein thrombosis without any local or apparent cause was what determined us to search further and investigate distant sites and rarer causes for this manifestation.
